# Temporospatial identification of language-related cortical function by a combination of transcranial magnetic stimulation and magnetoencephalography

**DOI:** 10.1002/brb3.317

**Published:** 2015-01-27

**Authors:** Misako Shinshi, Takufumi Yanagisawa, Masayuki Hirata, Tetsu Goto, Hisato Sugata, Toshihiko Araki, Yumiko Okamura, Yuka Hasegawa, Aya S Ihara, Shiro Yorifuji

**Affiliations:** 1 Department of Functional Diagnostic Science, Osaka University Graduate School of MedicineOsaka, Japan; 2 Department of Neurosurgery, Osaka University Graduate School of MedicineSuita, Japan; 3 Center for Information and Neural Networks (CiNet), National Institute of Information and Communications Technology, and Osaka UniversityKobe, Japan

**Keywords:** Event-related desynchronizations, inferior frontal gyrus, language lateralization, language processing, low gamma, picture naming

## Abstract

**Introduction:**

Identification of language-related cortical functions can be carried out noninvasively by transcranial magnetic stimulation (TMS) and magnetoencephalography (MEG), which allow for lesion-based interrogation and global temporospatial investigation of cortices, respectively. Combining these two modalities can improve the accuracy of the identification, but the relationships between them remain unclear. We compared TMS and MEG responses during the same language task to elucidate their temporospatial relationships and used the results to develop a novel method to identify language-related cortical functions.

**Methods:**

Twelve healthy right-handed volunteers performed a picture-naming task during TMS and MEG. TMS was applied on the right or left inferior frontal gyrus (IFG) at five time points, and the reaction times (RTs) for naming the pictures were measured. The temporospatial oscillatory changes measured by MEG during the same task were then compared with the TMS results.

**Results:**

Transcranial magnetic stimulation of the left IFG significantly lengthened RTs at 300 and 375 msec after picture presentation, whereas TMS of the right IFG did not change RTs significantly. Interestingly, the stimulus time point at which RTs increased significantly for each individual was correlated with when the low gamma event-related desynchronizations (ERDs) peaked in the left IFG. Moreover, combining the results of TMS and MEG improved the detection rate for identifying the laterality of language function.

**Conclusions:**

These results suggest that the low gamma ERDs measured by MEG strongly relate to the language function of picture naming in the left IFG. Finally, we propose a novel method to identify language-related cortical functions by combining TMS and MEG.

## Introduction

The temporospatial identification of language-related cortical functions is of great relevance across a wide spectrum of basic and clinical neuroscience (Tarapore et al. 2013). Various invasive and noninvasive methods have revealed that several cortical regions contribute to the processing of language function (Damasio and Tranel 1993; Warburton et al. 1996), such as the inferior frontal gyrus (IFG) (Berker et al. 1986; Koechlin and Jubault 2006) and the dorsal temporal lobe (Koechlin et al. 2003; Koechlin and Jubault 2006). Although previous research has shown that these cortical regions are generally essential for language functions, some individual differences exist in the temporospatial pattern of language-related cortical functions; for example, the laterality of the cerebral hemisphere corresponding to language function can vary among individuals (Ojemann et al. 2008; Bishop 2013). These individual differences may be problematic in the context of brain surgery, and reliable methods to identify the laterality of language function are needed (Hirata et al. 2010).

Recently, noninvasive investigation of cortical functions relating to language processing became possible (Deppe et al. 2004; Tarapore et al. 2013) with the advent of modern functional brain-imaging techniques such as magnetoencephalography (MEG) (Salmelin et al. 1994; Salmelin 2007), positron emission tomography (Petersen et al. 1988), and functional magnetic resonance imaging (functional MRI) (McCarthy et al. 1993; Medina et al. 2004). In particular, MEG provides precise temporospatial information about cortical activity during language processing (Martin et al. 1993; Salmelin et al. 1994) by using event-related potential (Papanicolaou et al. 1999) and frequency-dependent spatiotemporal modulation of cerebral oscillatory changes (Goto et al. 2011), such as an attenuation of the power in a frequency band, referred to as event-related desynchronizations (ERDs) (Pfurtscheller and Lopes da Silva 1999). Among several frequency bands, the ERDs of the beta and low gamma bands have been shown to be more valuable for identifying the laterality of language function (Hirata et al. 2004). We have already shown significant consistency between the laterality of ERDs and the Wada test (Hirata et al. 2010). However, the functional significance of these characteristic cortical activities for language processing is not fully understood.

Notably, MEG reveals not only the brain regions that are essential for language processing but also those that are nonessential; that is, “pseudopositive” areas. The problem of differentiating between the essential and nonessential areas can be avoided by the use of a suppression inspection method, such as the Wada test; direct electrical stimulation of cortices; and transcranial magnetic stimulation (TMS) (Crone et al. 2006; Wada and Rasmussen 2007). Although the Wada test and direct electrical stimulation are important methods for identifying the language-related cortical regions in neurosurgical patients to predict postoperative symptoms (Wada and Rasmussen 2007; Matsumoto et al. 2011), the invasiveness is disproportionate to its benefit, particularly with the recent advancement of noninvasive methods for human brain mapping (Pelletier et al. 2007). TMS is a promising alternative technique for noninvasive suppression inspection (Cowey 2005; Schuhmann et al. 2009). TMS transiently inhibits neural activity and can thus produce a measurable change in the performance of a task when applied to an area that is functionally and specifically involved in that task (Pascual-Leone et al. 2000). TMS was successfully applied to investigate the functional roles of the left IFG/Broca's area during language production (Schuhmann et al. 2009); however, its effect on language function remains controversial (Walsh and Cowey 2000). Moreover, it is not clear which cortical activities are affected by TMS during language tasks.

In this study, we conducted MEG and TMS experiments with the same language task to temporospatially identify the language-related cortical functions in healthy volunteers. The effects of TMS were compared with the temporospatial changes in the MEG responses to elucidate which oscillatory changes in MEG signals were the most critical for language processing. Moreover, we hypothesized that TMS and MEG are complementary techniques for precise identification of language-related cortical functions. To test our hypothesis, we compared how accurately the laterality of language function could be predicted using both modalities together versus each modality separately. On the basis of our results, we propose a novel noninvasive method for identifying language-related cortical functions by a combination of TMS and MEG.

## Methods

### Participants

Twelve healthy volunteers (two males and 10 females; mean age ± standard deviation [SD], 23.1 ± 3.3 years) participated in this study. All participants were strongly right-handed (as assessed by the Edinburgh Handedness Inventory; Oldfield 1971), had normal or corrected-to-normal vision, and had no history of neurological or psychiatric disorders. The experiments were conducted according to the principles of the Declaration of Helsinki, and the experimental procedures were approved by the Ethics Committee of Osaka University. Informed consent to participate in the study was obtained from all participants. No side effects were noted in any of the individuals during the course of the study.

### TMS experiments

#### Picture-naming task

**Figure 1:**
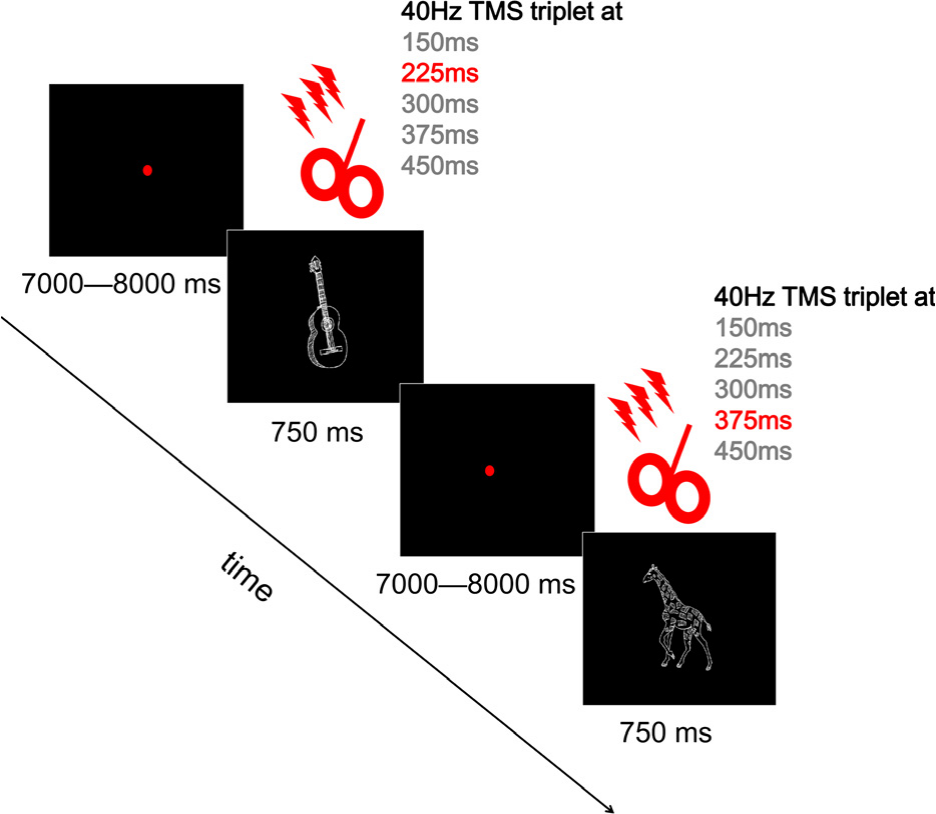
Experimental paradigm for transcranial magnetic stimulation (TMS). Each trial consisted of the presentation of a fixation point followed by the presentation of a picture. After picture presentation, TMS pulses were applied randomly at one of five different time points. Examples based on two trials are shown. In the first, the TMS pulses were applied 225 msec after the onset of the picture presentation, and in the second, the pulses were applied 375 msec after the onset of the picture presentation.

Participants performed a picture-naming task based on a previously reported paradigm (Schuhmann et al. 2009). Fifteen simple white-on-black line drawings were selected from those that were also used in MEG experiments. Each picture corresponded to a monomorphemic and monosyllabic three- or four-mora Japanese word selected from the NTT Database Series (Lexical properties of Japanese) with a word familiarity of 5–7 (Amano and Kondo 2000; Amano et al. 2007). The pictures were presented on a computer screen in front of the participant. The pictures subtended a horizontal visual angle of 3° and a vertical angle of 1°. Each trial consisted of a fixation point presented for 7000–8000 msec and presentation of one of the pictures for 750 msec (Fig.1). The duration of the fixation point was selected based on the previous studies (Schuhmann et al. 2009) and our preliminary observations. In the analysis, the beginning of the visual presentation of the picture was referred as time 0. Participants were instructed to name the presented picture as quickly as possible. The voices of participants were recorded with a digital electroencephalography (EEG) system (Neuro Prax; NeuroConn GmbH, Ilmenau, Germany) with a digital microphone positioned directly in front of the participant. The recorded voices were analyzed offline to identify onset timing of the naming by a sharp rising of the sound level and to evaluate the reaction times (RTs) for naming each picture. The whole experiment took about 1 h for each subject.

The TMS experiment consisted of two sessions. In one session, real TMS stimulation was used, and in the other session participants were subjected to sham TMS. The sequence of stimulation type was random across participants. Each session consisted of 60 trials, divided into four blocks of 15 trials for each. For each blocks, the same fifteen simple white-on-black line drawings were used. A visual presentation system (Presentation, Neurobehavioral Systems, Albany, CA, http://nbs.neurobs.com) was used to present pictures to subjects and to trigger TMS to start. Notably, there was no adverse effect of TMS such as the significant pain sensations elicited by the TMS. The TMS was conducted following the guideline for the use of TMS in clinical practice and research (Rossi et al. 2009).

Prior to starting the experiment, participants were familiarized with the stimuli and practiced naming the pictures repeatedly until stable RTs were achieved. RTs that were two SDs above or below the mean were defined as outliers and were excluded from analysis. Notably, the percentages of data defined as outliers were small: real TMS on left side, 5.27%; real TMS on right side, 4.3%; sham TMS on left side, 3.61%; and sham TMS on right side, 4.3%.

#### TMS conditions

Transcranial magnetic stimulation was applied with a Magstim Rapid stimulator (Magstim Co., Whitland, U.K.) and a figure-eight coil (D70 coil). The maximum outputs of this coil and stimulator combination were approximately 1.9 *T* and 150 A/*μ*sec. For sham stimulation, we used a specific figure-eight placebo coil to reproduce the acoustic stimulation of the active coil without inducing a magnetic field.

Event-related triple-pulse TMS was applied with an interpulse interval of 25 msec (40 Hz) at 150, 225, 300, 375, or 450 msec after the onset of picture presentation. The presentation of the pictures and the timing of TMS were fully randomized across trials within each session. RTs of left and right side were compared between the real and sham stimulations at each time point (Student's *t*-test).

For both real and sham stimulations, the coils were held manually, tangential to the skull, with the coil handle oriented perpendicular to the opercular part of the IFG, which was visualized using the online visualization function of the Brainsight navigation system (Rouge Research Inc., Montreal, QC, Canada) on the basis of a coregistered individual T1-weighted MR image. Stimulation sites for each subject were transformed into Talairach coordinates (Talairach and Tournoux 1988); the average coordinates across the subjects were *x* = 46.5 ± 3.2, *y* = 20.6 ± 3.4, *z* = 18.1 ± 3.9 for the right IFG and *x* = −47.6 ± 3.8, *y* = 23.4 ± 5.9, *z* = 19 ± 3.4 for the left IFG.

The stimulus intensity in real TMS sessions was set at 100% of the motor threshold. Individual motor thresholds were determined as the intensity at which stimulation of the left motor cortex with single-pulse TMS resulted in visible movement of the contralateral thumb. The motor thresholds of the participants ranged from 55% to 70% of maximum stimulator output (mean ± SD, 60.8 ± 3.6%).

#### Criteria to estimate laterality of language function using TMS

First, mean RTs of 12 participants for real stimulation were compared among both sides of IFG at the five time points to select the ones at which the mean RTs were significantly different between both sides (Student's *t*-test, Bonferroni corrected, *P* < 0.05). At these time points, either side of the IFG was assumed to have essential language functions. Then, to estimate the laterality of language function for each individual, changes in RTs between the real and sham stimulations were compared among both sides of IFG at each time point. A delay rate was calculated for each trial as 100 × ((RTs of real TMS for the trial) − (mean RTs of sham TMS))/(mean RTs of sham TMS). The delay rates were compared among TMSs on the right and left IFGs to determine the side on which the delay rates were significantly larger (Student's *t*-test, *P* < 0.05). For example, if delay rates of the left side significantly exceeded the delay rates of the right side (Student's *t*-test, *P* < 0.05), the laterality was inferred as being left. When the delay rates of both sides were not significantly different, the laterality was not estimated.

### MEG experiments

#### Picture-naming task

Each participant performed a picture-naming task during MEG measurement, and each session involved 100 simple white-on-black line drawings. Each picture corresponded to one monomorphemic and monosyllabic three- or four-mora Japanese word selected from the NTT Database Series (Lexical properties of Japanese) with a word familiarity of 5–7 (Amano and Kondo 2000). After the presentation of an eye-fixation point for 3000 msec, each picture was shown for 3000 msec. Participants were instructed to name each picture only once silently in their mind immediately after the presentation of the picture. The visual angles of the pictures were identical to those used in the TMS experiment. The whole experiment took about 1 h for each subject.

#### Measurement of neuromagnetic activity by MEG

Neuromagnetic activities were recorded in a magnetically shielded room with a 160-channel whole-head MEG system equipped with coaxial type gradiometers (MEG Vision NEO; Yokogawa Electric Corporation, Kanazawa, Japan). The participant lay supine on a bed with his or her head centered. Before and after recording, the head position was measured with five coils placed on the face (at the external meatus of each ear and at three points on the forehead). Pictures were displayed on a projection screen 325 mm from the participant's eyes by means of a visual presentation system (Presentation; Neurobehavioral Systems) and a liquid-crystal projector (LVP-HC6800; Mitsubishi Electric, Tokyo, Japan). Data were sampled at a rate of 1000 Hz and filtered with an online low-pass filter at 200 Hz. After data acquisition, a notch filter at 60 Hz was applied to eliminate AC line noise. To reduce contamination from muscle activities and eye movements, we instructed the participants to avoid shoulder movements and to watch the center of the display without moving their eyes or blinking. Some apparent artifacts were removed before the offline analysis.

#### Analysis of MEG data

The MEG data were analyzed by adaptive beam-forming, which is a narrow-band adaptive spatial filtering method (Sekihara et al. 2006). Estimates of differential source power between the control period and the period of interest for selected frequency bands and time windows were computed as pseudo-*T* values (Hirata et al. 2010). The control period was from 100 to 0 msec before the picture presentation onset. The periods of interest were selected as nine time windows of 100-msec sliding by 50 msec from 0 to 400 msec after the onset of the picture presentation. Statistical differences in power were compared between the periods of interest and the control periods for the following five frequency bands: theta (5–8 Hz), alpha (8–13 Hz), beta (13–25 Hz), low gamma (25–50 Hz), and high gamma (50–100 Hz) bands. The distribution of pseudo-*T* values was then superimposed on individual anatomical MR images coregistered to the MEG data. The larger pseudo-*T* values indicated the stronger ERD.

Individual statistical maps were generated by normalizing the beam-forming functional volumes to standard space for each time window and frequency band. First, a participant's MR image was coregistered with the MEG data by identifying the anatomical landmarks in the MR image that were used for placing the MEG head-localization coils (i.e., the left and right pre-auriculars and the nasion). The coregistered MR image was then spatially nonlinearly registered (normalized) to a template MR image using the SEG-toolbox in SPM8 (Litvak et al. 2011). Analysis at the voxel level was performed using a pseudo-*T* value incorporating variance smoothing with a 8-mm Gaussian kernel width. These individual statistical maps were then cut off by a threshold of *P* < 0.05 (corrected) and superimposed on the MNI template brain by means of MRIcron software (University of Nottingham School of Psychology, Nottingham, U.K., http://www.mricro.com).

Among temporospatial changes in each frequency bands, the low gamma ERDs for the left IFG were selected to characterize the cortical language function, based on the results of this study and our previous study (Goto et al. 2011). Low gamma ERDs in the IFG have been shown to estimate language laterality most consistently with the Wada test (Hirata et al. 2010). Moreover, our preliminary study showed that the TMS effects on language processing were stronger in the IFG compared to other areas. The peak pseudo-*T* value on each side of the IFG was evaluated at each time window for each subject. Then, the peak pseudo-*T* values were compared among the nine time windows by one-way analysis of variance (one-way ANOVA). Moreover, the mean pseudo-*T* values were compared between the right and left IFGs at each time window (Student's *t*-test, Bonferroni corrected). Finally, for each subject, we compared the peak time point of the low gamma ERDs and the time point at which the RTs of TMS on the left IFG significantly increased by real TMS compared to sham TMS using Spearman rank correlation coefficient (*r*_s_).

#### Criteria to estimate laterality of language function using MEG

Based on MEG measurement, laterality of language function was estimated from the laterality index (LI), which was calculated from the peak pseudo-*T* values of the ERDs for the left and right IFGs. The details of the definition of the LI and the protocol used to investigate functional laterality are described in a previous report (Hirata et al. 2004). Briefly, LI = 2(TR − TL)/(|TR| + |TL|), where TR and TL are defined as follows: the pseudo-*T* value of the most prominent ERDs in the low gamma band within the right IFG and that within the left IFG, respectively. The LI criteria for assigning the laterality of language function was as follows: when LI < −0.1, laterality was right sided; when −0.1 ≤ LI ≤ 0.1, laterality was bilateral; and when LI > 0.1, laterality was left sided.

#### Criteria to estimate laterality of language function using MEG and TMS

First, for each individual, a time window showing a peak of the low gamma ERDs in the IFG was selected. Then, at that time window, the delay rates were compared among TMSs on the right and left IFGs to determine the side on which the delay rates were significantly larger (Student's *t*-test, *P* < 0.05).

### MRI measurement

Anatomic MRI data were obtained with a 3.0-*T* whole-body MR scanner with a standard whole-head coil (Signa VH/i; GE Medical Systems, Milwaukee, WI). Individual MRI data consisted of T1-weighted sequences in 130 sagittal slices (1.4-mm thickness) with fiducial skin markers at the nasion and bilateral preauricular points.

## Results

### TMS-induced changes in response times during the picture-naming task

**Figure 2:**
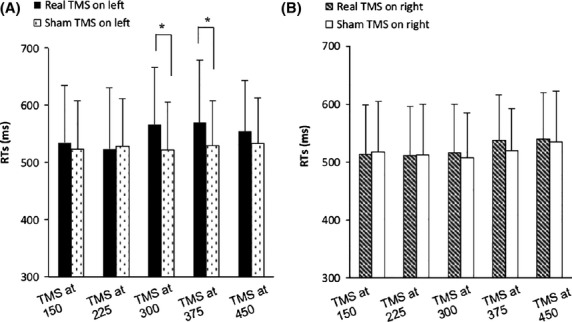
Mean reaction times (RTs) for real transcranial magnetic stimulation (TMS) (black) and sham TMS (dot) on the left (A) or right (B) inferior frontal gyrus (IFG) during the picture-naming task (*n* = 12). Asterisks indicate statistical significance of *P* < 0.05 (Student's *t-*test); error bars indicate standard deviations.

The RTs of real TMS and sham TMS on the right and left IFGs were tested at the five stimulus time points. For TMS on the left IFG, the mean RTs of real TMS was significantly longer than those of the sham TMS at 300 and 375 msec after the onset of picture presentation (Fig.2A) (Student's *t*-test, Bonferroni corrected, *P* < 0.05). In contrast, there was no significant difference in RTs of TMS on the right IFG at any stimulus time point (Fig.2B). Moreover, the RTs of real TMS on the left IFG were significantly longer than those of the real TMS on the right IFG at 300 and 375 msec (Student's *t*-test, Bonferroni corrected, *P* < 0.05). Notably, the point at which the RTs of TMS on the left IFG significantly increased with real TMS compared to sham TMS varied among participants; 300 or 375 msec for nine of 12 participants, 150 or 225 msec for 2, and none for 1.

### Low gamma ERDs during the picture-naming task

**Figure 3:**
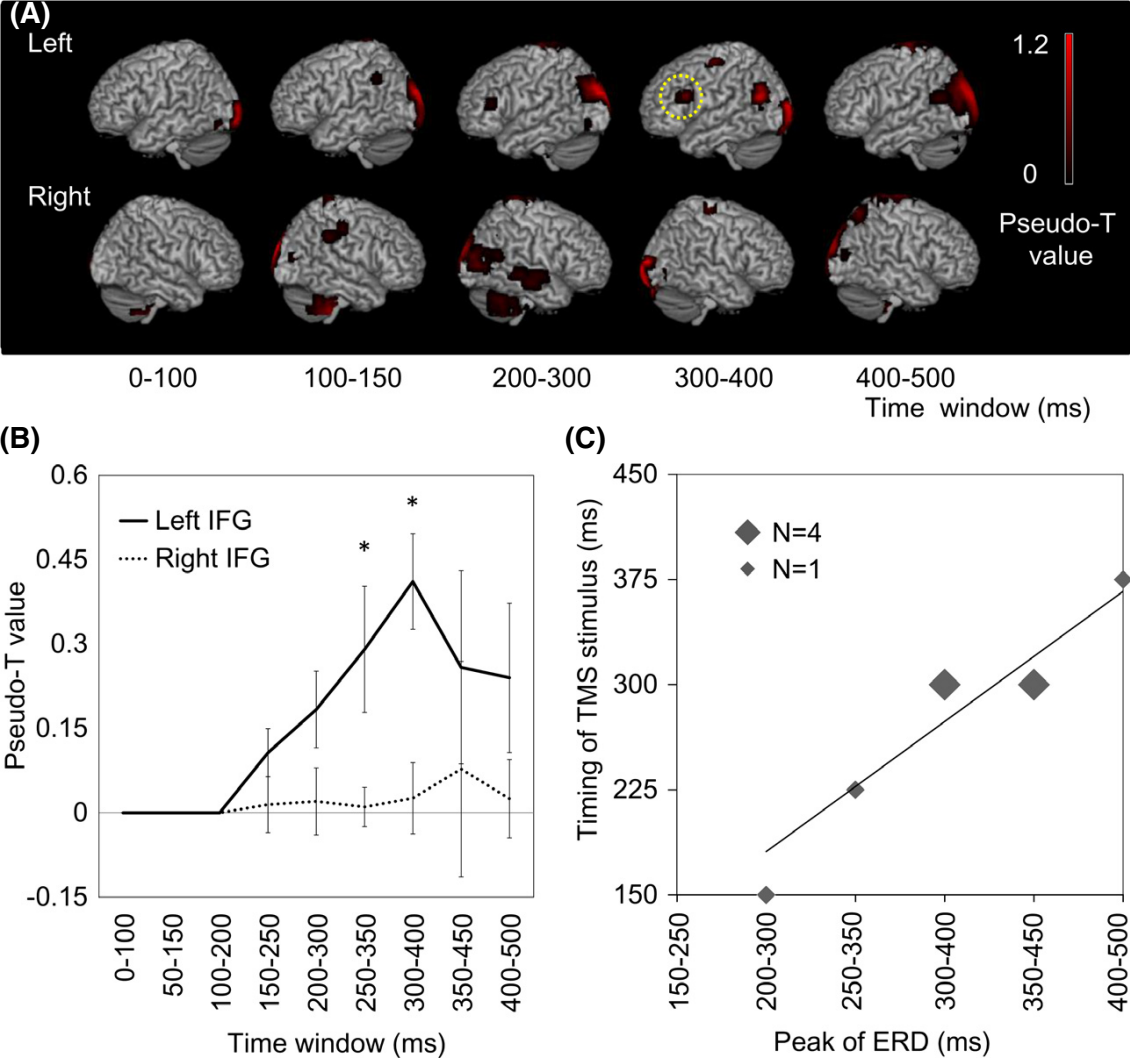
Temporospatial changes in the low gamma event-related desynchronizations (ERDs) during the picture-naming task. (A) A representative result of beam-forming analysis for subject 1. Pseudo-*T* values for low gamma ERDs are color coded on the reconstructed brain surface for each time window. The yellow circle indicates the peak pseudo-*T* value in the left inferior frontal gyrus (IFG). (B) The peak pseudo-*T* values of low gamma ERDs on the left or right IFG were averaged among subjects (*n* = 12) at each time window. The mean pseudo-*T* value of the left IGF was the highest at 300–400 msec (one-way ANOVA, *P* < 0.05) and was significantly larger than that of the right IFG at 250–300 and at 300–400 msec (*P < 0.05, Student's *t-*test). Error bars indicate standard error. (C) The time point of transcranial magnetic stimulation (TMS) at which reaction times (RTs) lengthened significantly was plotted at the peak of ERDs of the low gamma band. The size of the square indicates the number of patients included at the point. The stimulus timing at which RTs increased significantly was correlated with the time window during which ERDs peaked in the low gamma band. The correlation coefficient was 0.825 (*F* = 11, *P* < 0.01, Spearman's rank correlation). No correlation was observed for the other frequency bands.

A typical example of the temporospatial distribution of low gamma ERDs is shown in Figure3A. ERDs first appeared in the bilateral occipital cortices (Brodmann's area [BA] 17 and 18) in the 0- to 100-msec poststimulus window. Thereafter, the ERDs were observed at the bilateral middle temporal gyrus (BA 21), the bilateral supramarginal gyrus (BA 40), and the left IFG and the middle frontal gyrus (BA 44, 45, and 46). For other subjects, a similar pattern of temporospatial distributions of low gamma ERDs was observed. Notably, the low gamma ERDs on the left IFG was characteristically observed around 300 msec after picture presentation (indicated by the yellow circle in Fig.3A).

The time courses of the low gamma ERDs were compared between both sides of the IFG (Fig.3B) to clarify the temporospatial characteristics of the low gamma ERDs. The averaged pseudo-*T* value of the left IFG was the highest at 300–400 msec (one-way ANOVA, *P* < 0.05). And the pseudo-*T* value of the left IFG was significantly higher than that of the right IFG at 250–350 and 300–400 msec (Student's *t*-test, Bonferroni corrected, *P* < 0.05). The low gamma ERDs were found to be characteristically strengthened on the left IFG around 300 msec after picture presentation.

### Comparison of MEG and TMS results

For each subject, the peak timing of the low gamma ERDs was examined in relation to the time point at which the RTs of TMS on the left IFG significantly increased by real TMS compared to sham TMS. Interestingly, the peak timing of the pseudo-*T* value on the left IFG was concordant with the effective timing of the TMS on the left IFG. The stimulus time point of TMS on the left IFG at which RTs increased significantly between real TMS and sham TMS was significantly correlated with the time window during which the low gamma ERDs on the left IFG peaked (Fig.3C; *r*_s_ = 0.8264, *P* = 0.001788). In contrast, no such significant correlation was observed for the other frequency bands such as theta, alpha, beta, and high gamma band (*r*_s_ = 0.354 for beta, *r*_s_ = 0 for alpha, theta, and high gamma).

### Estimation of dominant hemisphere of language function by MEG and/or TMS

First, the dominant hemisphere of language function was evaluated using either MEG or TMS. As previously mentioned, the results of the TMS experiment showed that the RTs were significantly different between the real TMS and the sham TMS at 300 and 375 msec. Then, for both stimulus time points, the RTs of the real TMS and the sham TMS were compared for each individual. For nine of 12 participants, the RTs of real TMS exceeded the RTs of the sham TMS only for left side (Student's *t*-test, *P* < 0.05) and the delay rates of left side exceeded those of right side (Student's *t*-test, *P* < 0.05) to be evaluated as being left sided. For the remaining three participants, there were no significant differences in RTs between the real and sham TMSs. Therefore, the delay rates of each side were not significantly different for them (Table 1).

**Table 1:** Result of laterality by TMS and MEG

Participant no.	TMS	MEG	TMS and MEG
1	L^1^	L	L
2	L	L	L
3	L	L	L
4	L	L	L
5	L	L	L
6	L	L	L
7	L	L	L
8	–2	L	L
9	–	L	L
10	L	Bila^3^	L
11	L	L	L
12	–	R^4^	–

IFG, inferior frontal gyrus; MEG, magnetoencephalography TMS, transcranial magnetic stimulation.

1 Laterality was estimated as left sided. For TMS, the delay rates for left side were significantly larger than those for the right side. For MEG, the pseudo-*T* value of the left IFG was larger than that of the right side.

2 Laterality was not estimated (no significant difference of the delay rates between the two hemispheres).

3 Laterality was estimated as bilateral (no significant difference of pseudo-*T* value between the two hemispheres).

4 Laterality was estimated as right sided (the pseudo-*T* value of the right IFG was larger than that of the left side).

The LI, which was estimated by the low gamma ERDs in the IFG within the time window of 300–400 msec, showed the laterality of language function as left sided in 10 of 12 participants, bilateral in one, and right sided in one (Table 1).

**Figure 4:**
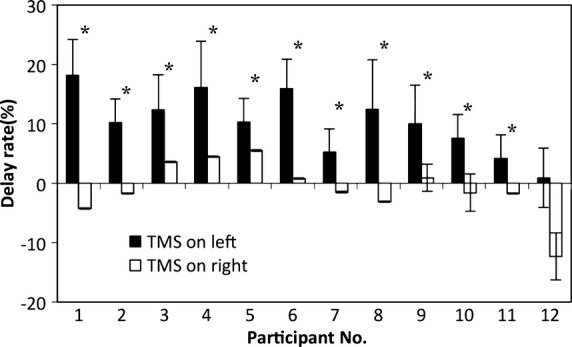
Delay rates of reaction times (RTs) were evaluated for all participants. The delay rates of either side of the inferior frontal gyrus (IFG) were averaged at the time that the low gamma event-related desynchronizations (ERDs) peaked in the IFG for each participant. Error bars indicate standard error. Asterisk indicates statistical significant of *P* < 0.05 (Student's *t-*test).

Next, a novel method combining TMS and MEG was applied to estimate the laterality. The delay rates of RTs on both sides of the IFG were compared at the time points at which the pseudo-*T* value of the low gamma ERDs reached a peak for each individual. That is, the RTs were evaluated at different time points for each individual depending on their low gamma ERDs. Using this method, 11 of 12 participants were determined to have left-sided laterality (Fig.4). Notably, the participant for whom language function was evaluated as being bilateral using MEG alone (no. 10) was evaluated as having left-sided laterality by using both MEG and TMS (Table 1). With the two modalities combined, the number of the participants determined to have left-sided laterality of language function increased.

## Discussion

In this study, we used TMS to evaluate the functional significance of temporospatial characteristics of language-related oscillatory changes in MEG during a picture-naming task. For each participant, RTs were significantly delayed when TMS was applied at the time point at which the low gamma ERDs on the left IFG were largest. This result suggests that low gamma ERDs occurred in a cortical area that was functionally essential for language processing. Moreover, by combining MEG and TMS, we precisely identified the language-related cortical regions with high temporospatial resolution, and we could therefore identify the laterality of language function. Our results demonstrate that combining MEG and TMS offers a novel method to evaluate the significant characteristics of language-related cortical functions.

### Time window for effective modulation of language function

Using TMS to modulate neural activity in the IFG, we identified the exact time point at which Broca's area was critically engaged in the process of picture naming. Specifically, real TMS on the left IFG significantly increased RTs compared to the sham TMS when the stimulus was applied at 300 or 375 msec after picture presentation. Therefore, the effective time window to modulate this picture-naming task was defined as 300–375 msec, which is compatible with previously reported estimates of the time course of language functions (Salmelin et al. 1994).

Some previous studies have estimated the temporal changes in speech production based on chronometrical behavioral and electrophysiological data (Indefrey and Levelt 2004). The access of a lexical concept, followed by the retrieval of the lemmas, takes approximately 200 msec (Thorpe et al. 1996; Schmitt et al. 2000). Schiller reported that phonological encoding and syllabification follow at approximately 350 msec and take approximately 100 msec (Schiller 2006). Phonetic encoding, the final step before articulation, takes approximately 145 msec. Based on the preceding studies, Levelt and colleagues indicated that the overall time required to name an object in a picture is between 550 and 600 msec (Levelt et al. 1998). In contrast to these other experiments and results, we obtained faster average RTs of approximately 500 msec. The faster times may be explained by our only using monomorphemic, monosyllabic words, and relatively familiar objects; naming these objects was the equivalent of a well-trained response. Presumably, the shorter RTs reflected a shortening of all the processes involved in speech production.

On the basis of that assumption, triple-pulse TMS at 300 or 375 msec after picture presentation onset is likely to disturb Broca's area in the process of syllabification. The timing differed between individuals, and TMS revealed the individual characteristics of language processing on a time scale of milliseconds.

### Temporospatial identification of language-related cortical activities

The frequency bands of MEG responses, such as alpha, beta, low gamma, and high gamma, have characteristic temporospatial modulations during language tasks (Salmelin et al. 1994; Goto et al. 2011). The band activities predominate in different cortical areas at different times; for example, low gamma ERDs predominate in the anterior part of the brain, beta ERDs in the middle part, and alpha ERDs in the posterior part (Goto et al. 2011). In a recent MEG study, we showed that during a silent reading task, ERDs first appear in the low gamma band in the 50–250 msec poststimulus window and that the temporal distributions of ERDs in the alpha, beta, and low gamma bands are parallel, and the broadest and highest ERDs occur in the left IFG at 200–400 msec after the stimulus (Goto et al. 2011). Our results for the temporal changes in ERDs corresponded to the previous study. Furthermore, we compared RTs at several time points and compared the ERD responses of each frequency band. We found that only the low gamma ERDs were correlated with significant modulation of RTs by TMS of the left IFG. These results suggest that the low gamma ERDs play an essential functional role in language processing. This finding is consistent with our previous results showing that lateralization and localization of low gamma ERDs in the frontal language areas are concordant with results obtained by the Wada test and cortical electrical stimulation mapping, respectively (Hirata et al. 2004, 2010). TMS of the left IFG may have an effect on language processing that is accompanied by low gamma ERDs.

Notably, in our study, high gamma activity was not shown to be related to the effects of TMS on language functions. However, because detecting activities in the high gamma band by MEG is difficult, the relationship between TMS and high gamma activities might be underestimated. Alternately, the high gamma band might be more associated with language function or processing than the low gamma band. Further investigation by means of an invasive method, such as electrocorticography, is necessary to elucidate the most important frequency bands for language function and to verify the relationship between high gamma and low gamma activities.

### Novel method to estimate the language lateralization

In this study, we found that the effective timing and location for real TMS corresponded to the peak of the low gamma ERDs measured by MEG. By combining TMS and MEG, we could detect language lateralization with greater sensitivity than we could with a single technique. Therefore, we propose the following novel method for evaluating language function based on combining MEG and TMS. First, MEG should be conducted to determine the time windows and brain areas at which the low gamma ERDs peaks, with the goal of determining the optimal conditions for TMS. TMS should then be conducted on the delineated time windows and areas at which ERDs peak. In this study, we were able to determine the laterality of the language-related area in 11 of 12 participants by combining TMS and MEG. We were also able to identify the laterality of language areas for one participant for whom laterality could not be determined by MEG alone. Notably, this result depends on the assumption that the laterality was left for all right-handed participants. To conclusively confirm this result, further studies are necessary to compare the proposed method with invasive techniques such as the Wada test (Pelletier et al. 2007). Still, the combination of the two different methods can be expected to allow more reliable and more highly sensitive evaluation and to serve as a noninvasive alternative to the Wada test.

## Conclusion

In this study, we used MEG in combination with TMS to evaluate the temporospatial characteristics of IFG activity related to language functions. Our results suggest that TMS on the left IFG affected cortical language functions accompanied by low gamma ERDs. By combining MEG and TMS, the individual differences in language function among the study participants were successfully evaluated.
